# Ultrawide thermal free-carrier tuning of dielectric antennas coupled to epsilon-near-zero substrates

**DOI:** 10.1038/s41467-017-00615-3

**Published:** 2017-09-07

**Authors:** Prasad P. Iyer, Mihir Pendharkar, Chris J. Palmstrøm, Jon A. Schuller

**Affiliations:** 1 Electrical and Computer Engineering Department, University of California Santa Barbara, Santa Barbara, CA 93106 USA; 2 Material Science and Engineering Department, University of California Santa Barbara, Santa Barbara, CA 93106 USA

## Abstract

The principal challenge for achieving reconfigurable optical antennas and metasurfaces is the need to generate continuous and large tunability of subwavelength, low-*Q* resonators. We demonstrate continuous and steady-state refractive index tuning at mid-infrared wavelengths using temperature-dependent control over the low-loss plasma frequency in III–V semiconductors. In doped InSb we demonstrate nearly two-fold increase in the electron effective mass leading to a positive refractive index shift (Δ*n* > 1.5) that is an order of magnitude greater than conventional thermo-optic effects. In undoped films we demonstrate more than 10-fold change in the thermal free-carrier concentration producing a near-unity negative refractive index shift. Exploiting both effects within a single resonator system—intrinsic InSb wires on a heavily doped (epsilon-near-zero) InSb substrate—we demonstrate dynamically steady-state tunable Mie resonances. The observed line-width resonance shifts (Δ*λ* > 1.7 μm*)* suggest new avenues for highly tunable and steady-state mid-infrared semiconductor antennas.

## Abstract (web summary)

Achieving large tunability of subwavelength resonators is a central challenge in nanophotonics. Here the authors demonstrate refractive index tuning at mid-infrared wavelengths using temperature-dependent control over the low loss plasma frequency in III-V semiconductors.

## Introduction

Optical antennas are subwavelength resonators with highly confined and enhanced electric fields that can be used to control the phase, amplitude and polarization of light^1, 2^. These resonators enhance the electric fields based on (1) plasmonic (metallic) resonances^3^ or (2) high-index low-loss Mie^4, 5^ (dielectric) resonances, and form the building blocks of numerous nanophotonic technologies. Individual resonators have been used to enhance performance of thermal emitters^6–8^, infrared detectors and sensors^9^, and resonator phased-arrays form the basis of various linear^1, 10^ and nonlinear metasurface components^11, 12^ Reconfigurable control over resonator properties is an existing challenge in any of these domains^13, 14^. Reconfigurable devices require shifting resonances (Δ*λ*_R_) by at least one line-width^15^ (*λ*_FWHM_) to extract sufficiently large amplitude^16^ or phase^17^ shifts. Achieving such tunability in resonators with inherently subwavelength dimensions and modest *Q*s^4, 18^, has motivated new approaches and designs for large-magnitude order-unity refractive index tuning (Δ*n* ≥ 1)^19^. For instance, large index shifts in phase change materials can enable switchable nanophotonic elements^20, 21^. For large-magnitude continuous tuning, many researchers have exploited free-carrier refraction at high carrier densities^22^.

Electrical depletion^23–25^ based devices can only achieve large index modulation over a few nanometers due to inherent tradeoffs between depletion width and carrier density. As a result, such schemes have only demonstrated small resonance shifts (Δ*λ*_R_ << *λ*_FWHM_). Larger shifts (Δ*λ*_R_ > *λ*_FWHM_) are possible in free-carrier injection (electrically^17^ or optically^12^ pumped) based devices. The short lifetimes in these far-from-equilibrium devices is useful for ultra-fast operations^15, 26^, but makes steady-state operation challenging and highly power-consuming. Here, we demonstrate a thermal free-carrier refraction effect, where we tune the plasma frequency in InSb with temperature by changing both the electron mass and density. Exploiting the small bandgap and highly non-parabolic band structure of InSb we produce refractive index shifts (Δ*n* > 1.5) far greater than conventional thermo-optic shifts. Further we experimentally demonstrate thermally driven steady-state resonant shifts (Δ*λ*_R_ ~ *λ*_FWHM_) of dielectric (i-InSb) antenna resonators strongly coupled to highly doped (n-InSb) epsilon-near-zero (ENZ) substrates.

## Results

### Thin film refractive index shifts

InSb forms an ideal thermally tunable system due to its low bandgap (*E*_g_ = 0.17 eV at 300 K), low conductivity effective mass ($m_{\rm{e}}^*{\rm{ = }}0.014{m_0}$ at the conduction band minimum at 300 K)^27^ and high refractive index (*n* = 4). Through moderate doping (10^16^–10^19^), the infrared permittivity of InSb can be continuously engineered between high refractive index (*ε*_R_ ~ 16) and plasmonic (*ε*_R_ < 0) regimes according to simple Drude models^28^.

1 $$\varepsilon = {\varepsilon ^\prime } + i{\varepsilon ^{\prime \prime }}{\rm{ = }}{\varepsilon _\infty }\left( {1 - \frac{{\omega _{\rm{p}}^2}}{{{\omega ^2} + i\,\Gamma \omega }}} \right);\,\omega _{\rm{p}}^2 = \frac{{{N_{{\rm{free}}}}{e^2}}}{{{\varepsilon _{\rm{o}}}{\varepsilon _\infty }m_{\rm{e}}^{\rm{*}}}}.$$

**Fig. 1:**
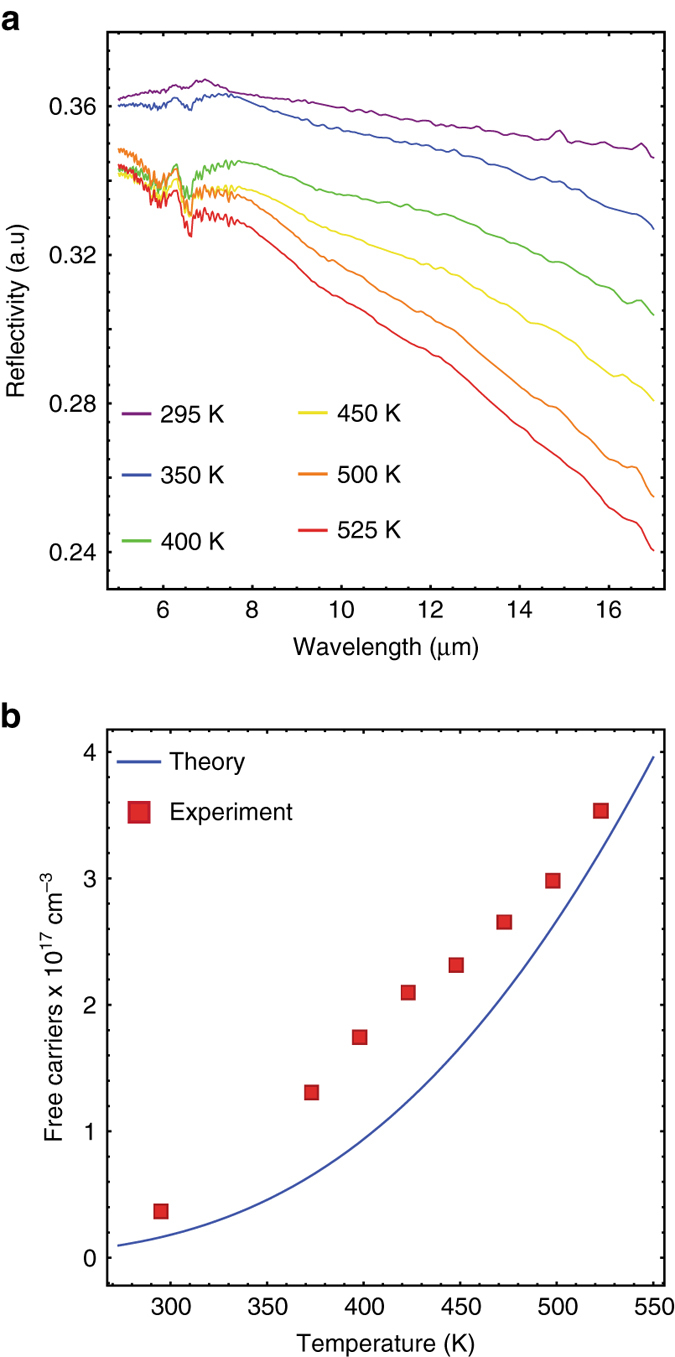
Thermal free-carrier refraction in intrinsic InSb. **a** Reflection spectra of an intrinsic InSb film at various temperatures. The long-wavelength roll-off is due to Drude dispersion and increases with temperature. **b** Temperature-dependent free-carrier concentrations inferred from fits to the curves in Fig. 1a (*square red dots*) show good agreement with fit-free models of thermally activated carriers (*blue*)

Exploiting this tremendous control over the IR optical properties, researchers can design “mesoscale” metamaterials where dielectric and metallic properties are seamlessly integrated within a single-material system via molecular beam epitaxial (MBE) growth techniques^29, 30^. As an example of dynamic control of these IR optical properties, consider the experimental reflectivity curves from an intrinsic (i.e., undoped) InSb wafer shown in Fig. 1a. At room temperature (*purple*) the reflectivity is nearly constant with wavelength as expected for a simple dielectric interface with refractive index *n* ~ 4. The drop in reflectivity at long wavelengths is consistent with a thermally generated free-carrier density of ${n_{\rm{i}}} \cong 4 \times {10^{17}}{\rm{c}}{{\rm{m}}^{ - 3}}$. As the temperature is increased, the magnitude of the long-wavelength reflectivity roll-off increases as well. By fitting these reflectivity curves (Supplementary Note 1) we extract the electron concentration (Fig. 1b) at each temperature. Note that the fits incorporate established models for the temperature-dependent band minimum electron effective mass (Eq. 2, where *α* = 3.9 eV^−1^ is the band non-parabolicity from the Kane model^31–33^). The extracted electron densities (*red squares*) closely match theoretical^34^ predictions (Eq. 3):

2 $$m_{\rm{e}}^{\rm{*}}\left[ T \right] = m_0^{\rm{*}}\sqrt {1 + 4\alpha kT} $$

3 $${n_i}[T] = 2.9 \times {10^{11}}{\left( {2400 - T} \right)^{0.75}}\left( {1 + 2.7 \times {{10}^{ - 4}}} \right){T^{1.5}} \\ \exp \left( { - \frac{{0.129 - 1.5 \times {{10}^{ - 4}}T}}{{{\rm{k}}T}}} \right) \cdot \qquad$$

**Fig. 2:**
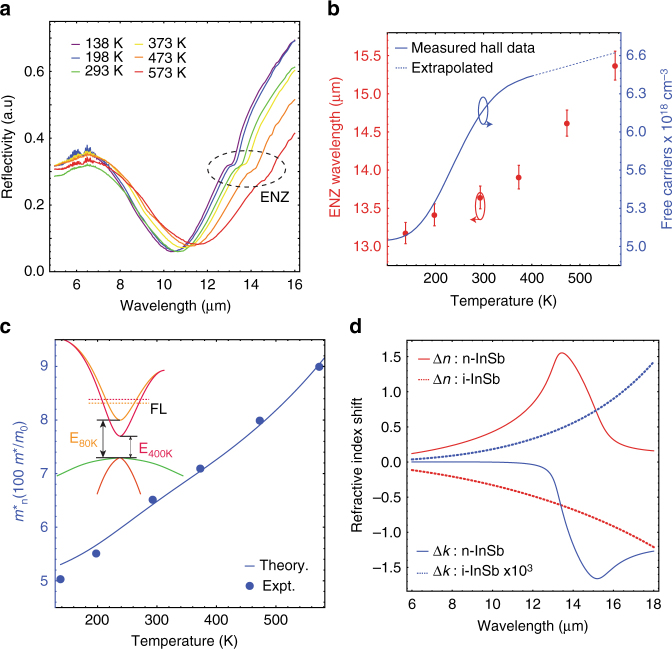
Refractive index modulation of doped InSb. **a** Reflection spectra of a highly doped InSb film at various temperatures. The *dashed ellipse* highlights a feature on the curve that marks the epsilon-near-zero (*ENZ*) wavelength. The ENZ wavelength red shifts with increasing temperature. **b** Plot showing the thermal dispersion of *λ*_ENZ_(*red dots*, *left axis*) and free-carrier concentration (*blue curve*, *right axis*). *λ*_ENZ_ is determined from transfer-matrix modeling of plots in Fig. 2a and the *error bars* represent possible variation in the measurements due to the detector resolution limit. Free-carrier concentration is determined from Hall measurements. **c** Plot showing the experimentally determined optical effective mass (*m*_optical_, *blue dots*) as a function of temperature. The effective mass increases by ~80%. The *blue curve* (*left axis*) indicates the predicted thermal dispersion of the electron effective mass for n-InSb according to Eqs. 4, 5. (inset) As temperature increases, changing band curvature and a shrinking bandgap leads to effective mass variation at the Fermi-level (*dashed lines*) for highly doped InSb **d** Plot showing the fitted refractive index change for both doped InSb (real part: *red solid line*; imaginary part: *blue solid line*) between 573 K and 138 K and intrinsic InSb (real part: *red dashed line*; imaginary part: *blue dashed line*) from 295 K to 525 K. Note that the real part (imaginary part) increases (decreases) when doped InSb is heated, while the opposite is true for intrinsic InSb

At long wavelengths, a 230 K change in temperature can effect a larger than unity change in the refractive index (Fig. 2d) with a nearly negligible impact on losses (Δ*k* < 2 × 10^−3^). Note that the origin of this effect is fundamentally different than traditional thermo-optic effects in semiconductors; consequently the magnitude ($2n\frac{{\partial n}}{{\partial T}} \cong - 0.02$ at *λ* = 13.5 μm) is more than an order of magnitude larger than thermo-optic shifts reported for any group IV or III–V semiconductors^35^ (Supplementary Table 1). This large reduction in refractive index with temperature is in marked contrast to the behavior of doped InSb, described below.

The ability to engineer low-loss ENZ materials by doping III–V semiconductors has enabled various applications^36–40^. However, steady-state tuning of ENZ wavelengths in the bulk has not yet been demonstrated. ENZ substrates consisting of 1.2 μm thick heavly n-doped (Te dopant of 5 × 10^18^ cm^−3^) single-crystal InSb films were MBE grown on lattice matched i-GaSb substrates (Methods). Temperature-dependent reflectivity curves are shown in Fig. 2a. The reflectivity curves exhibit reflection minima at the onset of the plasma edge^41^ (*ε*_R_ ~ 1) followed by a longer wavelength bulk plasmon^42, 43^ at the ENZ wavelength^44^. As the sample is heated, both features shift to longer wavelengths, indicating a reduction in the Drude weight and a corresponding increase in refractive index. The ENZ wavelength shifts by up to ~20% (Fig. 2b, *red symbols*), providing a new approach for generating tunable ENZ phenomena. Fitting reflectivity curves with a transfer-matrix method incorporating Drude models (Supplementary Note 1), we extract the scattering rate (Γ) and plasma frequency *ω*_p_ at each temperature. Unlike intrinsic InSb, where *ω*_p_ increases with temperature, the red-shifting features in Fig. 2a are indicative of a decreasing plasma frequency.

To understand the origins of this decrease in plasma frequency we perform temperature-dependent Hall measurements to independently measure the electron density (*blue curve*, Fig. 2b) and subsequently infer the elecron effective mass. Unlike the order of magnitude changes in carrier density for i-InSb, the carrier density for the doped films is relatively constant with temperature. The red-shift in plasma wavelength can only be explained by an increase in the electron effective mass. In heavily n-doped InSb, the Fermi-level lies well above the conduction band minimum and the non-parabolic band strucutre plays a significant role. (Supplementary Note 2) For instance, the extracted low-temperature (*T* = 138 K) effective mass (0.05) is more than three times larger than the value at the conduction band minimum (0.014*m*_0_ at 300 K). As the temperature is increased to 573 K the effective mass increases by ~80% (*Blue circles*, Fig. 2c). The inferred effective mass shows excellent agreement with a theoretical model^28, 45^ (*blue line*, Eq. 4) developed here to account for the temperature-dependent bandgap (Eq. 5 for *T* < 600 K)^46–48^, band curvature (Eq. 2), and Fermi distribution:

4 $$m_{\rm{n}}^{\rm{*}}[T] = m_{\rm{e}}^{\rm{*}}[T]\sqrt {1 + \frac{1}{2}{{\left( {\frac{3}{\pi }} \right)}^{\!\!\frac{2}{3}}}\frac{{{{\rm{h}}^2}\,{n_{{\rm{free}}}}{{[T]}^{\frac{2}{3}}}}}{{e{E_{\rm{g}}}[T]\,m_{\rm{e}}^{\rm{*}}[T]}}} $$

5 $${E_{\rm{g}}}\left[ T \right] = {10^{ - 3}}\left( {235 - \frac{{0.27{T^2}}}{{T + 106}}} \right)$$

The wavelength dependent change in the complex refractive index upon heating (138–573 K) the n-doped InSb films is plotted in Fig. 2d. The real part of the refractive index increases by a maximum of 1.5 while the imaginary part of the refractive index decreases by 1.75 due to the increasing electron mass and accompanying reduction of the Drude response. The imaginary part of the dielectric constant varies between 0.5–0.7 at the ENZ wavelength across the whole temperature range, making it comparable with other low-loss ENZ materials^49, 50^. The increase in losses arises from increased free-carrier scattering due to dopant activation. The magnitude of the refractive index change shown in doped InSb is orders of magnitude larger than conventional thermo-optic effect demonstrated in traditional semiconductors (Supplementary Table 1). These results demonstrate a large (80%) dynamic variation of electron effective mass with temperature and exploiting such effects for refractive index (Δ*n* > 1.5, Δ*k* > 1.7) modulation. We also show that fit parameter-free model (Eq. 4) developed to include the temperature variations closely matches the experimentally measured electron effective mass. The model of temperature-dependent effective mass developed here closely matches experiments and thus clarifies inconsitencies^28, 33, 45, 51^ in the literature regarding the temperature-dependent electron effective mass in n-InSb. Within a single-material system we thus observe two different plasmonic tuning mechanisms: changing the free-carrier concentration (intrinsic) or electron effective mass (doped). Below, we show that these distinct mechansims can alternately be used to achieve large resonance red shifts or blue shifts within a single resonator geometry.

The resonance wavelength of nano-antennas can be tuned by changing the refractive index of the resonator or of a supporting substrate^52, 53^. Here, we show that i-InSb wire resonators fabricated on top of doped InSb layers demonstrate both classes of tunability, corresponding to the two distinct plasmon tuning mechanisms demonstrated above. Specifically, individual resonators exhibit (1) transverse electric (TE) resonances that are heavily coupled to an ENZ substrate and (2) transverse magnetic (TM) high-index Mie resonances that are almost independent of the underlying substrate. The distinct nature of these two resonances provides distinct temperature-dependent tuning capabilities.

### Geometric dispersion of dielectric antennas on ENZ substrate

**Fig. 3:**
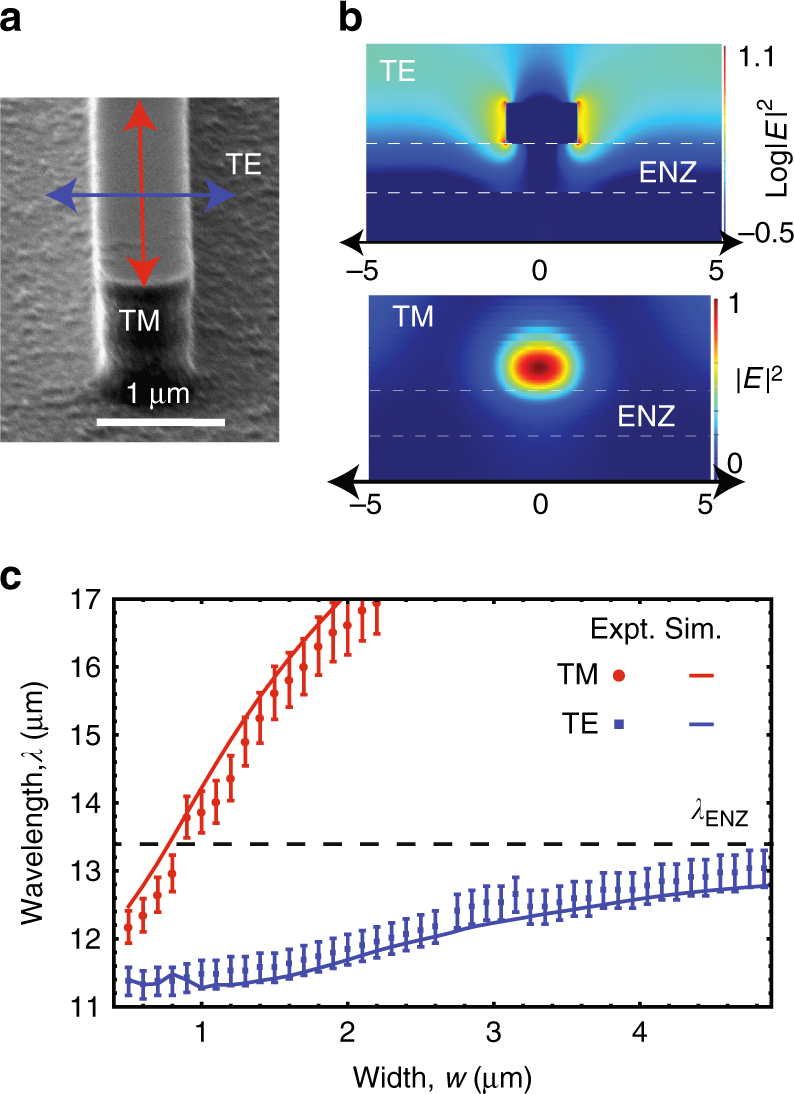
Geometric dispersion of mie resonators on ENZ substrate. **a** Scanning electron microscopy (*SEM*) images of the etched intrinsic InSb resonators of height 1 µm, length 500 µm (long side) and widths 700 nm (short side) on the doped substrates. **b** Plot showing the resonant electric field intensity profiles under TE-polarized and TM-polarized illumination (w.r.t the short side) of a 1 μm wide resonator. The electric field intensity is concentrated outside the resonator, at the substrate-resonator edge for the TE-polarized mode while the resonant fields are almost completely localized inside the resonator for the TM-polarized mode. **c** Plot showing the experimentally measured geometric dispersion of the TE (*blue squares*) and TM (*red dots*) resonances as function of resonator width and the *error bars* represent possible variation in the measurements due to the detector resolution limit. The *solid lines* (TE: *blue* and TM *red*) show the FDTD simulated peaks in scattering cross section of single resonators. The TE resonance exhibits a far weaker geometric dispersion and does not cross the ENZ wavelength of the substrate (*black dashed line*), indicating a highly substrate-coupled mode

Tunable resonators comprise low-loss dielectric (i-InSb, Fig. 3a) wires (width *w* of 500 nm to 5 µm, 500 µm long, and 1 µm tall) fabricated on heavily n-doped InSb films (Methods). TE-polarized and TM-polarized resonances of individual structures (Fig. 3a) are measured with an FTIR microscope. The reflected signal from a single resonator is normalized to an equivalent area background (substrate of same area of 50 × 500 µm and polarization)^22^. Experimentally measured dips in normalized reflection spectra (*circles*) show excellent agreement with scattering peaks in finite difference time domain (FDTD) simulations (*lines*) for single resonators of varying widths (Fig. 3c) (Supplementary Note 2). These resonances correspond to equivalent magnetic (TM_0_) and electric (TE_1_) Mie resonances of an infinite cylindrical^54^. TM (*red*) and TE (*blue*) polarized resonances show markedly different geometric dispersions. For the smallest width structures TE and TM resonances occur at similar wavelengths, where the substrate index is approaching zero. As width (*w*) increases (for a fixed height), TM modes show a strong dispersion of wavelength whereas TE modes exhibit far weaker size dispersion. For high-index cylindrical Mie resonators, the ratio of resonance wavelengths $\left( {\frac{{{\lambda _1}}}{{{\lambda _2}}}} \right)$ is exactly equal to the square root of the ratio of cross-sectional area $\left( {\sqrt {\frac{{{A_1}}}{{{A_2}}}} = \sqrt {\frac{{{w_1}}}{{{w_2}}}} } \right)$. A similar trend is expected for variations near square cross-section (*w* = 1.2 μm), and is consistent with the TM mode (8% increase in $\sqrt w$ → 6% change in *λ*_res_) but totally inconsistent with the TE mode (100% increase in $\sqrt w$ → 13.2% change in *λ*_res_).

These differences in the geometric dispersion can be understood through an examination of the accompanying resonant field intensity profiles (Fig. 3b). The TM resonant electric fields are almost completely localized within the resonator. The overlap of the electric field with the substrate is minimal and the geometric dispersion is almost independent of the substrate refractive index. On the other hand, the TE resonant electric fields are concentrated outside the resonator and are strongly coupled to the ENZ substrate. Similar to plasmonic resonators on ENZ substrates^42, 55, 56^, the mode thus exhibits a highly dispersive effective index *n*_eff_(*λ*). The resonance wavelegnth is proportional to the product of the width and effective index (*λ*_*res*_ ∝ *n*_eff_ *w*); increasing width is offset by a decreasing *n*_eff_ and the geometric dispersion is small with resonances getting “pinned” near the ENZ wavelength, an effect previously seen only in plasmonic resonators. The difference between these two modes becomes particularly evident when we examine the temperature-dependent wavelength shifts.

### Thermal dispersion of dielectric antennas on ENZ substrate

**Fig. 4:**
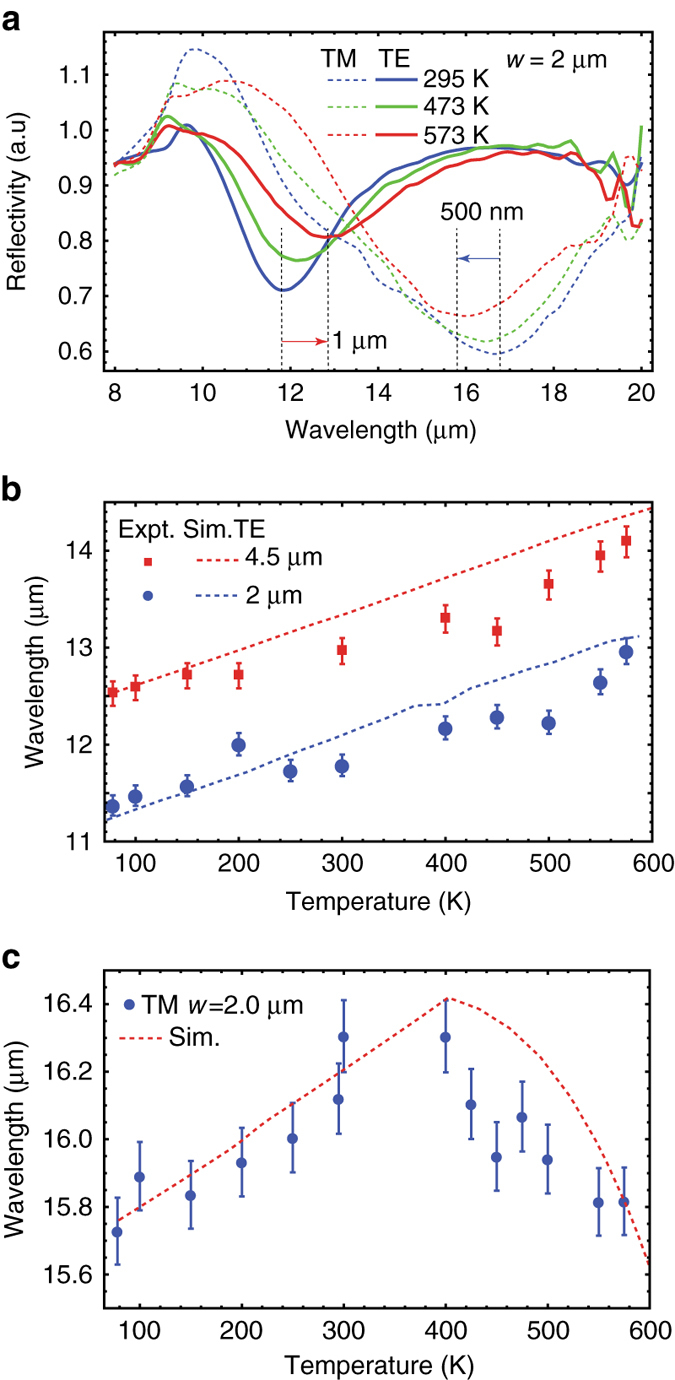
Thermal dispersion of TE vs TM resonances. **a** TE (*solid*) and TM (*dashed*) reflection spectra of a 2 µm wide resonator at different temperatures (295 K: *blue*, 473 K: *green* and 573 K: *red*). The TM resonance red shifts while the TE resonance blue shifts with increasing temperature. **b** Thermal dispersion of the experimentally measured (*dots*) and FDTD simulated (*dashed lines*) TE resonance as a function of temperature for 2 µm (*blue*) and 4.5 µm (*red*) wide resonators. The resonances shift linearly by 1.5 µm from 80 K to 573 K for both resonators, in close agreement with FDTD simulations. **c** Thermal dispersion of the TM resonance for a 2 µm wide resonator. The resonance initially red shifts slowly due to substrate effects. Beyond 400 K free-carrier concentration increases rapidly leading to a reduced refractive index of the resonator and accompanying resonance blue shifts. Both regimes of tunability are well accounted for in simulations (*red dashed line*). The *error bars* in **b**, **c** represent possible variation in the measurements due to the detector resolution limit

Examples of temperature-dependent reflection spectra for 2 μm wide resonators on ENZ substrates are shown in Fig. 4a. The TE resonance (*solid lines*) red shifts with heating while the TM resonance blue shifts. The TE resonance shifts are dominated by substrate refractive index changes; the increasing effective mass in the substrate (decreasing Drude weight) red shifts the ENZ wavelength. The TE modes are pinned near this wavelength and red-shift in turn. The TE mode shifts agree well with simulations, exhibiting roughly linear resonance shifts across a large temperature range (80–573 K) independent of resonator size (Fig. 4b). This dynamic tuning of the resonant wavelength is broadband (11–14 µm) around the ENZ wavelength tuning range while the full width half max of the resonant dips broadens with the increase in the scattering losses (0.5–0.7) at the ENZ wavelength. The speed of tuning for such thermal effects is limited by slow thermal time scales. However, since the electron effective mass depends in part on the position of the fermi-level within the conduction band, large resonance shifts can potentially be driven electrically^17^ at far faster time scales. Even though the TE modes are broad $\left( {Q = \frac{{{\lambda _{\rm{R}}}}}{{\delta {\lambda _{{\rm{FWHM}}}}}} \sim 2.5 - 6} \right)$, the large-magnitude index shift enables tuning by a line-width.

The TM resonances, on the other hand, exhibit more complex behavior across the full temperature range of these studies. The mode initially red shifts at low temperatures, then demonstrates very rapid blue shifts at temperatures above ~400 K (Fig. 4c). At low temperatures, the change in i-InSb free-carrier concentration with heating is minimal. The small, observed red shifts are mainly due to changes in the finite, negative permittivity of the doped substrate. At higher temperatures (*T* > 400 K), thermally generated free-carrier concentrations become significant ( > 10^17^ cm^−3^) and the resonator refractive index decreases quickly. Measurements of i-InSb on other substrates are expected to display larger shifts due to a lack of competition between substrate and resonator effects. Regardless, both effects are well accounted for in temperature-dependent simulations (*dashed line*), which show good agreement with experimental results.

## Discussion

**Fig. 5:**
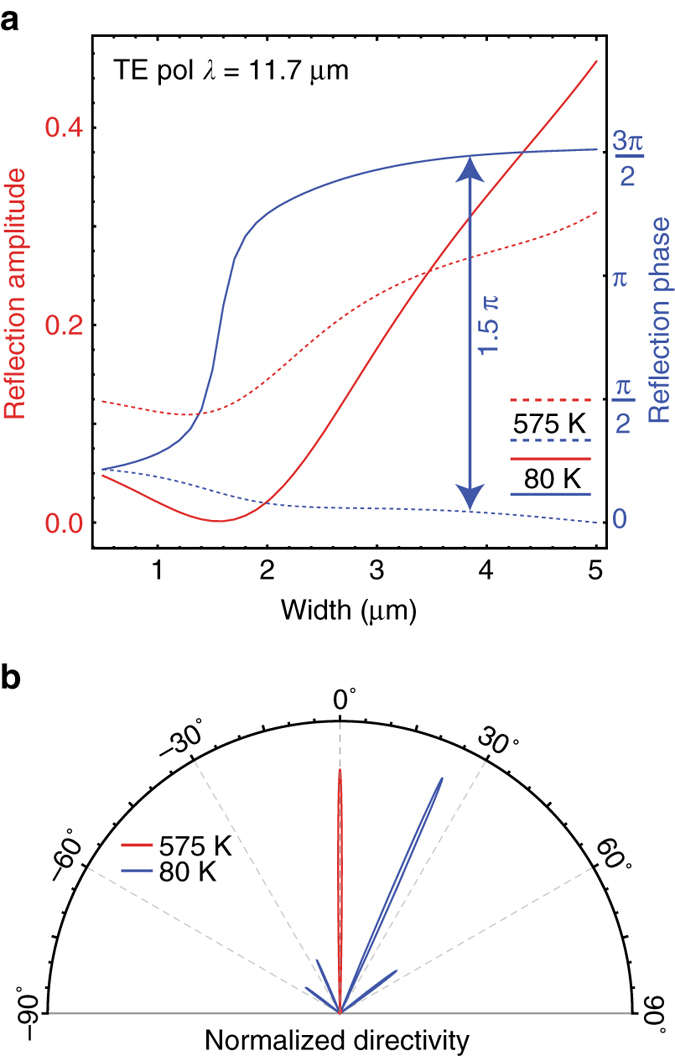
Thermal beam-steering metasurface. **a** Width-dependent reflection amplitude (*red*, *left axis*) and phase (*blue*, *right axis*) from a periodic array (6 μm periodicity) of 1 μm tall InSb wires at a wavelength of 11.7 μm. At 575 K (*dashed*) the phase is nearly constant with width. Due to pinning of the TE resonances, this evolves into a 3π/2 size-dependent phase variation at 80 K (*solid*) **b** Using these phase functions, a reconfigurable metasurface can be designed with normal incidence reflection at 575 K (*red*) and a unidirectional diffraction lobe at 80 K (*blue*). The polar plot shows the normalized directivity of the (10 unit cell) metasurface which reflects a normally incident light towards 25° at 80 K (*blue*) and 0° at 575 K (*red*)

Large-magnitude thermal free-carrier tuning may be used to construct various reconfigurable optical antenna and metasurface devices. For instance, spatially uniform temperature modulation can be used to dynamically tune otherwise static metasurface properties formed through geometric phase engineering. For instance consider the width-dependent TE reflection phase of InSb resonators shown in Fig. 5. Due to the unique “pinning” effect of the TE resonance, a nearly size independent reflection phase at 575 K (*dashed blue*) evolves into a large (~3 π/2) size-dependent phase dispersion at 80 K (*solid blue*). In a beam-steering device (Fig. 5b) this effect can be used to switch a beam-steering lobe from 0° to 25° through uniform heating. While more research is needed to optimize the potential of thermal free-carrier tuning, these results highlight the possibility of using this effect in a variety of steady-state temperature-controlled mid-infrared metadevices.

Within a single-material system, large refractive index shifts can be generated by either tuning of the electron effective mass (substrate tuning, visible in TE modes) or density (resonator tuning, visible in TM modes). These effects lead to a diversity of behaviors within even a single resonator geometry and are well characterized by simulations that incorporate temperature-dependent Drude models. The observed large-magnitude (Δ*n* > 1) refractive index shifts are far larger than conventional thermo-optic effects and suggest new avenues for highly tunable and reconfigurable mid-infrared Mie resonators, ENZ materials, and metasurfaces.

## Methods

### Growth and fabrication

Molecular Beam Epitaxy growth of the layer structure (GaSb substrate/1.2 μm Te-doped InSb/1 μm i-InSb) was performed using a modified VG-V80H MBE system with a base pressure <5 × 10^−11^ Torr. The undoped GaSb substrate was thermally desorbed under an Sb overpressure, after which a thin buffer of GaSb was grown. The substrate temperature was lowered to less than 380 °C for the growth of Tellurium doped InSb, which was followed by growth of undoped InSb at the same growth temperature. After MBE growth, a 300 nm thick film of SiO_2_ was deposited using plasma-enhanced chemical vapor deposition to form a hard mask for subsequent dry etching. Photolithography was performed using a projection stepper aligner with SPR 90 as the photoresist (PR) along with contrast enhancing mixture top layer to increase verticality of the developed PR. The hard mask was etched using an inductively coupled plasma dry etch using CHF_3_/Ar gas mixture followed by a 10 min Oxygen plasma clean at 250 °C to remove any organic residue from the sample surface. The InSb was etched using a reactive ion etching process using a methane plasma and timed to stop at an etch depth of 1 μm. The excess hard mask was removed using a wet etch with a buffered HF solution for 60 s.

### Hall measurement

The temperature-dependent hall measurements were performed on the MBE grown structure containing doped and undoped layers of InSb from 2 K to 400 K using a Quantum Design Physical Property Measurement System in a Van-der-Paw geometry using a standard AC lock-in technique.

### FDTD simulation

The scattering cross section simulations for the single resonators were performed in a commercially available FDTD Solutions (Lumerical Inc.) software using the inbuilt cross section monitor and total-field-scattered field source. The symmetric and asymmetric boundary conditions were employed for TM and TE polarization simulations to accelerate the FDTD convergence. The two-dimensional simulations of wire cross section showed exceptional matching with the measured 3D wire resonator and the meshes around the resonator and ENZ substrate were minimized (1×1 nm) to field divergence due to the vanishing dielectric constant. The near-field monitor was used to map the electric field intensity at the resonant wavelengths.

### Data availability

All data sets supporting the findings of this study are included in this published article (and its Supplementary Information files). Further data sets are available from the corresponding author on reasonable request

## Electronic supplementary material

Supplementary Information
