# Supplementary material for: Ultrawide thermal free-carrier tuning of dielectric antennas coupled to epsilon-near-zero substrates
Source: Nat Commun. 2017 Sep 7;8:472. doi: 10.1038/s41467-017-00615-3 (PMC5589832; doi:10.1038/s41467-017-00615-3)
Supplement: Supplementary file 1 — Supplementary Information [file 41467_2017_615_MOESM1_ESM.pdf]

Description of Supplementary Files

File Name: Supplementary Information
Description: Supplementary Figures, Supplementary Table, Supplementary Notes and Supplementary References

Supplementary Note 1: Transfer Matrix Model Fitting for the Drude parameters

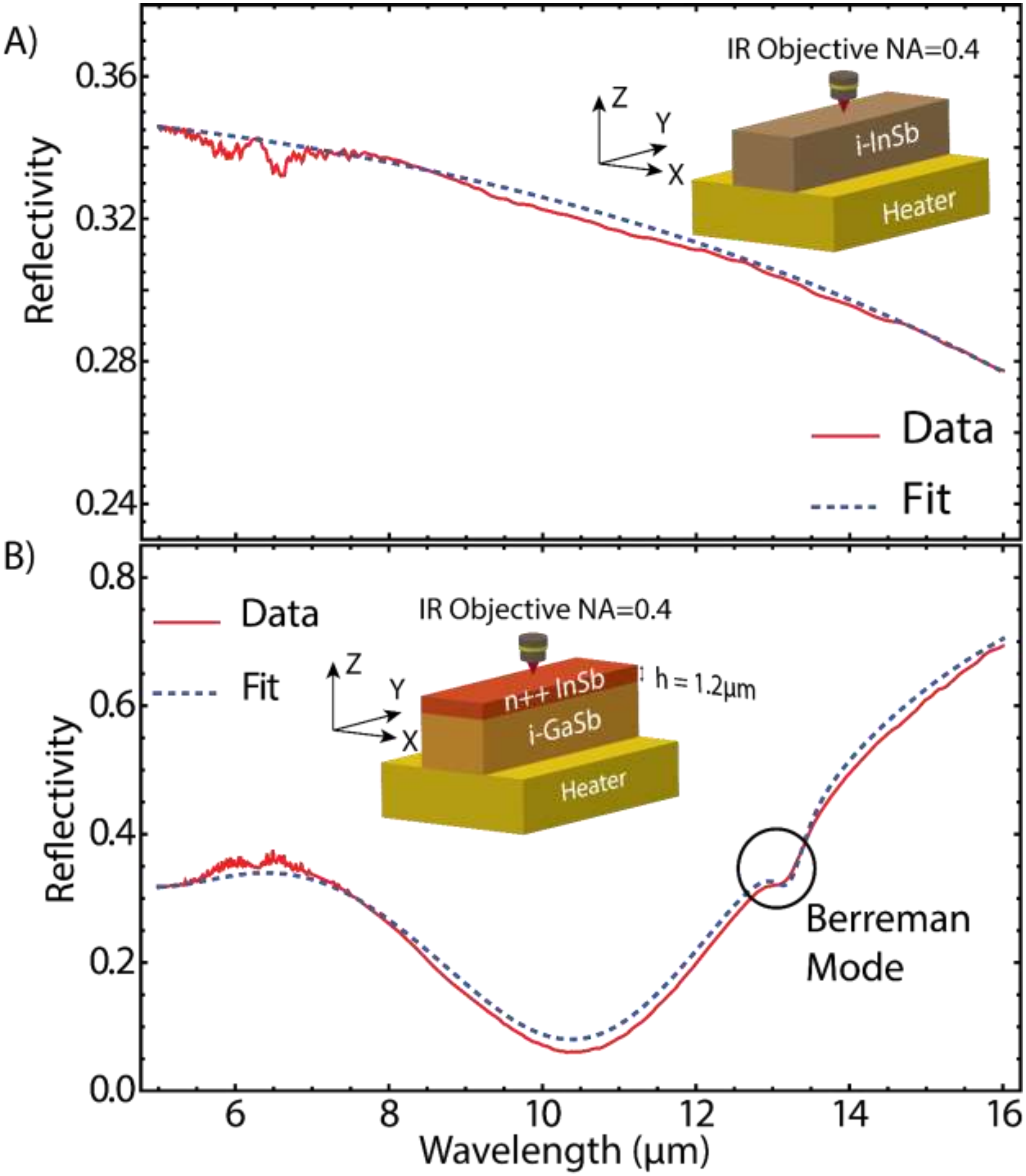

Supplementary Figure 1| Fitting reflectivity curves: A) Reflectivity spectrum from single crystal substrate of intrinsic InSb (Red, solid) and the analytical Fresnel reflection fit curve for an infinite InSb slab (Blue, Dashed). The reflectivity rolls off at long wavelengths due to Drude dispersion from thermally activated carriers **B)** Reflectivity spectrum from a thin film of doped

InSb on GaSb (Red, solid) and the Transfer matrix fit curve for the same stack under off-normal incidence (Blue, Dashed). The small "kink" in the data around 13.5μm, highlighted by the black circle, shows the Berreman mode near the ENZ wavelength. The inset sketch shows the measurement setup.

The intrinsic InSb reflectivity curves (normalized to Au) are fit based on simple Fresnel equations for a semi-infinite slab of dielectric whose refractive index model includes free-carrier Drude dispersion. Reflectivity roll off at higher wavelength is caused by thermally-generated free-carriers throughout the whole substrate. From these fits we determine the electron density as a function of temperature. The Mid-infrared reflection spectra (normalized to Au) of doped InSb is fit to a transfer matrix model of a single dielectric film on a semi-infinite slab of GaSb. The reflection spectra exhibits distinct characteristics that enable unique fits of the Drude (equation 1) plasma frequency (ω_p) and scattering rate (Γ) . The dip in the reflection at 11μm marks the plasma edge (n~1) while the "kink" on the rising edge of the reflection curve at 13μm marks a Ferrel-Berreman mode near the ENZ wavelength (n~0). The peak in the reflection curve 6μm enables us to determine the thickness of the thin film (1.2μm), assuming GaSb substrate index at 3.8 and ε_∞ of InSb at 15.68. As the plasma frequency increases the plasma edge dip blue shifts. Reflection measurements are performed in an Infrared microscope with 15X objective, ensuring off-normal incidence angles up to ~20°. This enables coupling to the Ferrel-Berreman bulk plasmon mode of the thin film. Fits of the scattering rate (Γ) are primarily sensitive to the slope of the rising edge and sharpness (or line-width) of the Ferrel-Berreman feature. As the scattering rate increases, the slope of the rising reflectivity curve for λ > plasma edge dip decreases along with the line-width of the kink. Thus the thermal dispersion of the plasma frequency and the scattering rate are determined with little correlation between the two parameters. Using measured free carrier concentration from temperature dependent Hall measurements, the electron effective mass can be determined uniquely.

Supplementary Note 2: Geometric Dispersion of the resonances from FDTD.

The scattering cross-section from a single wire resonator is measured using power-monitor outside the total field scattered field (TFSF) plane wave polarized source. The dips in measured reflection curves match closely with the peaks in simulated cross section curves (Fig 3B). There is a slight offset for TE (red shift) and TM (blue shift) polarized resonances measured due to a substrate effect introduced through the normalized spectral measurements.

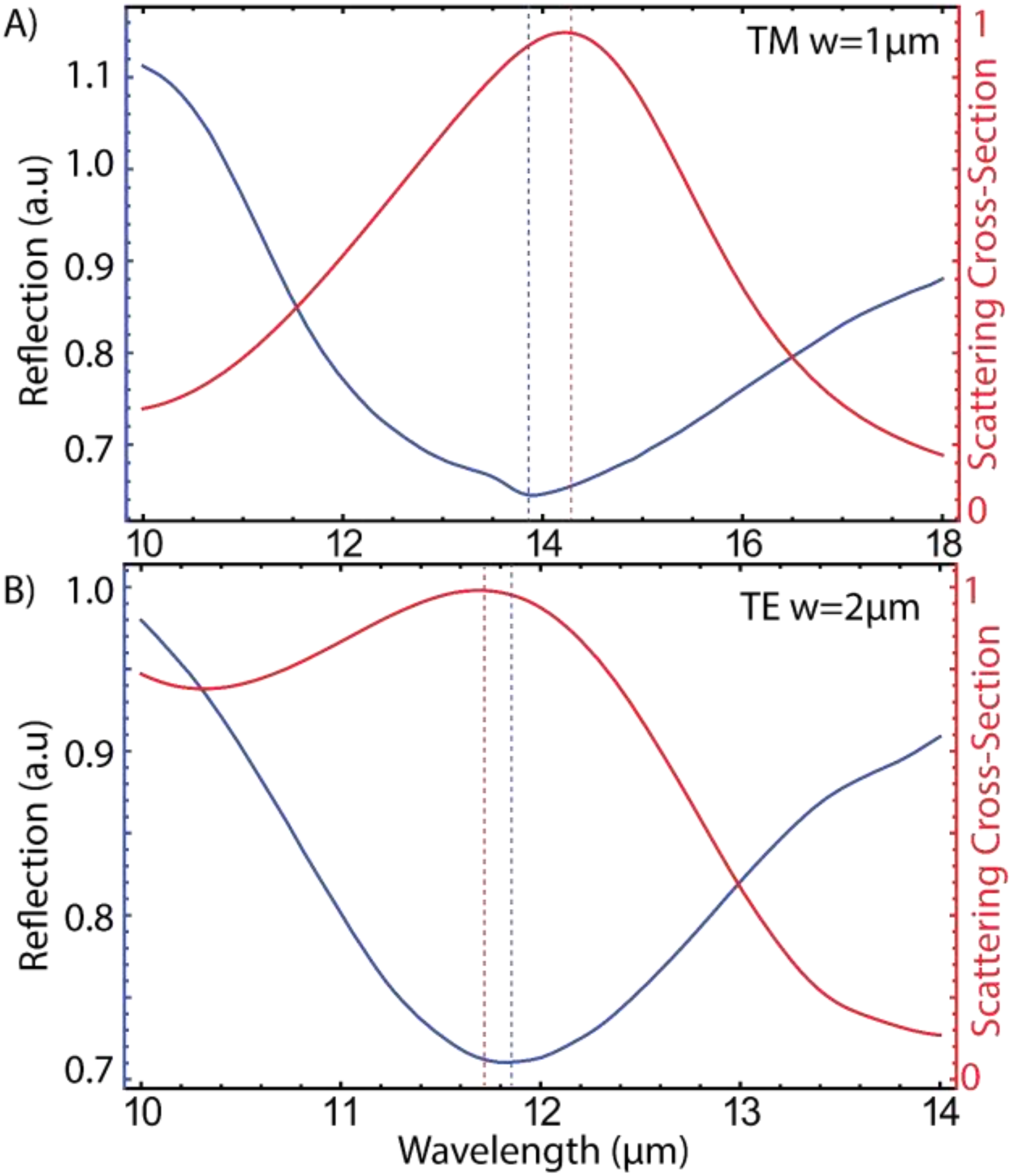

Supplementary Figure 2|Peak matching between reflection plots with FDTD simulations: A) Experimentally measured reflectivity curve (Blue, left axis) and simulated normalized scattering cross-section curve (Red, right axis) under TE polarization for a resonator of width 1µm **B)** Experimentally measured reflectivity curve (Blue, left axis) and simulated normalized scattering cross-section curve (Red, right axis) under TM polarization for a resonator of width 2µm.

Supplementary Note 3: Comparing Thermo-plasmonic shift of InSb with traditional thermo-optic shift

Semiconductor	$\frac{\partial \epsilon}{\partial T} \approx 2n\frac{\partial n}{\partial T} \times 10^{-4}/K$	Ref
Si	11.6	1
Ge	33.3	1
InAs	21.8	1
InSb*	37.3	1
GaSb	22.9	2
InP	6.75	2
GaAs	18	2
PbSe	-90	3
PbTe	-145	3
i-InSb**	$-224+1.3\times10^{-3}\,i$	This work
n-InSb***	$385-14.3i$	This work

Supplementary Table 1| Thermo-optic Coefficients for Traditional Semiconductors in the LWIR wavelength: The table shows the thermo-optic coefficient of different semiconductors in the 10-15µm wavelength range.*Traditional thermo-optic coefficient measured below 200K at 10.6µm **Intrinsic InSb thermo-plasmonic shift reported in this work is based on the thermal free carrier generation based refractive index shift at 13.5µm between 350K and 525K. ***The thermo-plasmonic co-efficient of the doped InSb reported is based on the electron effective mass shift. The thermo-plasmonic coefficient values for i-InSb and n-InSb reported in the table has a quadratic chromatic-dispersion based on the Drude model described in the main text.

Supplementary References

1. Gillen, G. D., DiRocco, C., Powers, P. & Guha, S. Temperature-dependent refractive index measurements of wafer-shaped InAs and InSb. *Appl. Opt.* **47,** 164 (2008).
2. Yu, P. Y. & Cardona, M. Temperature Coefficient of the Refractive Index of Diamond- and Zinc-Blende-Type Semiconductors. *Phys. Rev. B* **2,** 3193–3197 (1970).
3. Ghosh, G. *Handbook of Thermo-Optic Coefficients of Optical Materials with Applications. Academic Press* (Academic Press, 1998).
